# Dehydration With Non-diarrheal Illnesses in Pediatric Patients: Insights From Real-World, Electronic Medical Records Retrospective Data

**DOI:** 10.7759/cureus.107539

**Published:** 2026-04-22

**Authors:** Harshad Malve, Rahul Nagpal, Pavitra Chakravarty, Amol Patil

**Affiliations:** 1 Medical Safety Sciences, Kenvue, Mumbai, IND; 2 Pediatrics, Flt. Lt. Rajan Dhall Fortis Hospital, New Delhi, IND; 3 Pediatrics, Bhagirati Neotia Mother and Child Hospital, Kolkata, IND

**Keywords:** children, dehydration, electronic medical records, fever, oral electrolytes, real-world data

## Abstract

Introduction

Diarrheal dehydration is well known with specific guidelines. However, non-diarrheal dehydration is often unreported. To date, there has not been a single study to report the prevalence of dehydration due to non-diarrheal conditions in pediatric patients.

Materials and methods

Electronic medical record (EMR) data from January 2017 to March 2023 for patients with non-diarrheal illnesses meeting inclusion and exclusion criteria were retrieved and analyzed to understand the prevalence of dehydration due to non-diarrheal conditions in Pediatric patients. Further, the associated conditions for these patients with dehydration were also evaluated.

Results

Of the total, 18,89,680 pediatric patients, merely 1,888 patients (0.09%) had dehydration-related complaints recorded in the prescription. The percentage was strikingly less than the expectations from real-world data. Respiratory tract infections (RTIs) and fever were observed to be the most common associated non-diarrheal conditions for patients with dehydration.

Conclusion

There is a need to bridge the reporting gap by creating awareness among pediatricians and physicians to manage dehydration in non-diarrheal illnesses appropriately for better patient outcomes.

## Introduction

Infants and young children are more susceptible to developing dehydration due to their higher metabolic demands, inability to alert caretakers to their hydration needs, and increased insensible water loss [1]. Dehydration is commonly observed in pediatric patients, including but not limited to diarrheal conditions. Fever, influenza, urinary tract infections (UTI), bronchiolitis, and gingivostomatitis [2-4], nausea, vomiting, heat exhaustion, viral illnesses, and respiratory tract infections (RTI) are the other conditions that lead to fluid electrolytes (FE) deficits in children [5]. There are well-defined guidelines for the management of dehydration due to diarrhea. However, in the absence of formally laid guidelines for managing dehydration due to non-diarrheal conditions, available guidelines for diarrheal conditions are often extrapolated to the non-diarrheal conditions [6,7]. Physicians usually prescribe oral rehydration salts (ORS) for the management of diarrheal dehydration; the powder format of it needs reconstitution in water for consumption, where the usual errors take place. While for the non-diarrheal conditions, the patients’ needs may vary, and hence electrolyte drinks can be used. Therefore, in such conditions, the solutions should contain adequate electrolytes and some energy along with fluid [7].

Clinicians assess hydration status by correlating history and different signs and symptoms presented [8,9]. There are numerous studies highlighting the prevalence and treatment of dehydration in patients with diarrhea. However, dehydration due to non-diarrheal conditions is a less studied topic, and to date, no existing data highlighting the prevalence of dehydration in non-diarrheal conditions is available. The present study was planned to bridge this gap by analyzing the real-world data. The primary objective of this study was to evaluate the prevalence of dehydration in non-diarrheal conditions in pediatric patients (<18 years) in the real world and understand the associated conditions.

## Materials and methods

Study design

This was a retrospective, real-world, electronic medical record (EMR)-based study.

Data source

Anonymized, aggregated patient-level data of pediatric patients from January 2017 to March 2023 who met the eligibility criteria were retrieved from HealthPlix EMR (https://healthplix.com).

Population definition

As per the defined age criteria, pediatric patients (<18 years) who were diagnosed with non-diarrheal illnesses like fever, infections, nausea, vomiting, tropical fever (e.g., dengue, malaria, chikungunya) were included in the study. Patients with the mentioned complaints as free text in the EMR, such as diarrhea, loose motions, loose stools, increased frequency of bowel movements, acute gastroenteritis, and other related terms mimicking diarrhea in their prescriptions, were excluded from this study. Additionally, patients diagnosed with cardiac failure, renal failure, ascites, liver cirrhosis, or pancreatitis were also excluded. The data on demographic characteristics [age/sex], symptoms, diagnosis, duration of illness, and daily recommendation of oral electrolyte formulations (e.g., WHO oral rehydration salts (ORS), electrolyte drinks like ORSL, Johnson & Johnson; Enerzal, Mankind Pharma Ltd; Prolyte, Cipla Ltd, etc.) were retrieved and analyzed. The patients who have “dehydration” mentioned that the complaints or diagnosis section of the EMR is marked as “dehydrated”. The study solely depends on the data captured on EMR and hence may have selection bias, along with the limitation of missing data.

Ethics

The protocol for this study was reviewed and approved by the Royal Pune Independent Ethics Committee (No. RPIEC041123, dated November 2, 2023). Owing to retrospective analysis of the EMR data, this study was not registered on the Clinical Trials Registry - India portal.

Statistical analysis

Statistical analysis for this study was carried out using Stata version 15.1SE (StataCorp LLC, College Station, TX, USA**)**. Pediatric patients with non-diarrheal illnesses with reported symptoms of dehydration, and associated conditions of those patients were presented using frequency, and percentage.

## Results

Patient disposition and prevalence of dehydration

**Figure 1:**
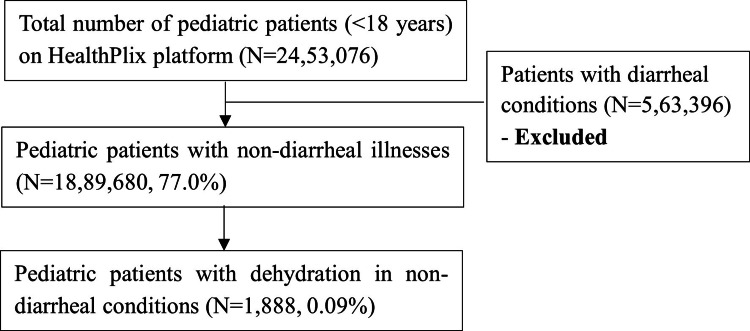
Patient disposition and prevalence of dehydration

There were 24,53,076 pediatric patient records available in the HealthPlix EMR database from January 2017 to March 2023. Amongst them, 18,89,680 (77.0%) patients were diagnosed with non-diarrheal conditions. The 5,63,396 (23%) patients with diarrheal conditions were excluded from the study. Out of the 18,89,680 patients with non-diarrheal conditions, only 1,888 (0.09%) patients were mentioned to be dehydrated as per the records in Figure 1.

Year-wise patient data is summarized in Table 1.

**Table 1:** Year-wise patient data

Number of patients	2017 (Jan-Dec) n (%)		2018 (Jan-Dec) n (%)	2019 (Jan-Dec) n (%)	2020 (Jan-Dec) n (%)	2021 (Jan-Dec) n (%)	2022 (Jan-Dec) n (%)	2023 (Jan-Mar) n (%)	Total n (%)
Total patients on HealthPlix, n		161572	998299	2849989	4107880	6967670	10119254	4139633	22490726
Pediatric patients (<18 years), n (% of total patients)		8457	52997	255299	330141	636200	1188373	493657	2453076
		(5.2)	(5.3)	(9.0)	(8.0)	(9.1)	(11.7)	(11.9)	(10.9)
Non-diarrheal illnesses, n (% of pediatric patients)		7471	42548	200904	263452	501541	918027	384414	1889680
		(88.3)	(80.3)	(78.7)	(79.8)	(78.8)	(77.3)	(77.9)	(77.0)
Dehydration cases, n (% of non-diarrheal cases)		1	15	93	126	299	976	435	1888
		(0.01)	(0.03)	(0.04)	(0.05)	(0.06)	(0.10)	(0.11)	(0.09)

Associated conditions

**Figure 2:**
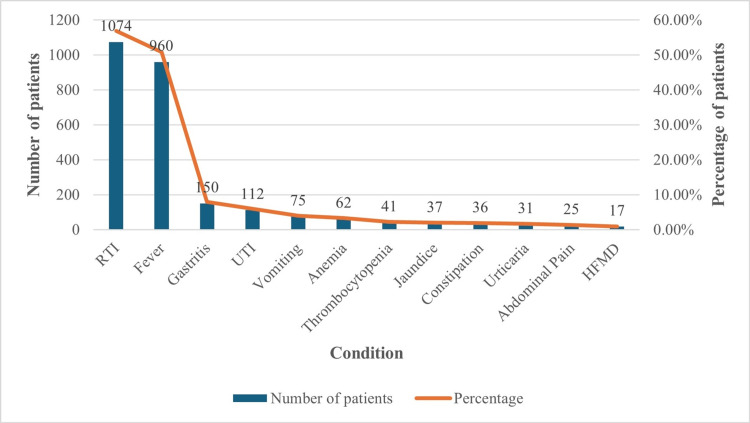
Pediatric patients with non-diarrheal illness having symptoms of dehydration and its associated conditions HFMD: hand foot mouth disease, UTI: urinary tract infection, RTI: respiratory tract infection

We assessed the concomitant conditions reported for pediatric patients with dehydration. RTI and fever were the commonly reported conditions for 57% and 51% of the patients, respectively. Gastritis, UTI, and other conditions reported for pediatric patients are presented in Figure 2.

All the associated conditions are summarized in Table 2. 

**Table 2:** Associated conditions for pediatric patients with dehydration Patients can have one or more conditions.

Associated conditions	Overall (N=1888) n (%)
Respiratory tract infection	1074 (56.89)
Fever	960 (50.85)
Gastritis	150 (7.94)
Urinary tract infection	112 (5.93)
Vomiting	75 (3.97)
Anemia	62 (3.28)
Thrombocytopenia	41 (2.17)
Jaundice	37 (1.96)
Constipation	36 (1.91)
Urticaria	31 (1.64)
Abdominal pain	25 (1.32)
Hand foot mouth disease	17 (0.90)

## Discussion

Dehydration due to non-diarrheal illnesses like fever, tropical fevers, nausea, vomiting, etc., often goes unreported. Patients with dehydration often complain of fatigue and general weakness. Recommendations for taking oral electrolyte drinks in such conditions might result in better patient compliance and outcomes in comparison to recommendations of solid food during the phase of anorexia [6]. The present study was conducted to study an under-researched area prevalence of dehydration in non-diarrheal illnesses in the pediatric patient population.

It was observed from the real-world data that a very small proportion of patients had dehydration reported in their records. One of the reasons for this low reporting could be the practice of not proactively recording dehydration as a complaint, or doctors are not proactively assessing dehydration. A recent study by the Indian Council of Medical Research (ICMR) reported that there were deviations in 45% of the prescriptions, and 9.8% of them had unacceptable deviations [10]. This low prevalence of dehydration observed from the data can also be due to underdiagnosis since there is no concrete test to identify it, and clinicians have to rely on the history, signs, and symptoms presented [11]. Doctors do not explicitly mention dehydration in the medical records or prescriptions. The study suggests that clinicians do not follow prescription practices when stating diagnosis, including dehydration owing to non-diarrheal diseases. It has been reported that the need for prevention and treatment of dehydration in such conditions is generally overlooked. However, it should be recognized that adequate levels of fluids and electrolytes are crucial for maintaining the health and function of vital organs. Fluid requirements in children are known to be higher in comparison to adults because of a higher caloric expenditure and more insensible water losses, specifically in infants [5,12]. Also, body water percentage is higher in children with higher body surface area as compared to adults. Children cannot communicate their hydration requirements, which may lead to fluid and electrolyte deficits.

In our study, RTI and fever were observed to be the most associated non-diarrheal conditions in patients with dehydration. This is consistent with earlier clinical studies and available literature. Factors like the patient’s body temperature, respiratory rate, body surface area, and overall fluid intake are known to have a big impact on insensible water losses. Patients with acute RTI are often advised to increase their fluid intake to overcome the insensible fluid loss due to tachypnoea [13]. Paul et al. mentioned that patients with bronchiolitis, a viral RTI, present with features of fever, coughing, wheezing, dehydration, and nasal discharge [14]. In case of fever, insensible losses are known to increase by more than 10% with a per degree Celsius rise in the body temperature [15]. A study by Boutin et al. was reported to understand the association between dehydration and fever in the first week of life [16]. The authors concluded that in the pediatric emergency department, dehydration was commonly associated with fever in infants who were less than eight days of age [16].

In this study, UTI was also commonly associated with dehydration. Johnson et al. reported that underhydration is often associated with UTIs [17]. It is recommended that increased water intake could result in increased urine output, potentially reducing the risk of UTIs [17]. It is inferred that reporting dehydration is very low in clinical settings. Efforts should be made to adopt good prescribing practices, including complete practices. This will also help with the effective management of conditions. This study includes an extensive analysis of a huge database of patients in the form of EMR. Being a retrospective, EMR-based data, the inherent limitation is that only available data was captured and was available for analysis. This leads to the possibility of missing data, which was not recorded in the EMR. Raw data is available for any further scrutiny or analysis. HealthPlix operates only from private outpatient settings, mostly from urban and semi-urban locations, and does not cover hospital records and emergency departments. Therefore, the cases of severe dehydration requiring hospital admissions and treatment are not covered. The data from electronic medical records or prescriptions captures the recorded data by healthcare professionals (HCPs), and the possibility of incomplete data.

## Conclusions

The prevalence of dehydration in pediatric patients with non-diarrheal illnesses was 0.09%, which appears lower considering the susceptibility of children to dehydration during these illnesses. There is a need for proactive screening, recognition and documentation for dehydration in children with non-diarrheal conditions.
